# Editorial: Rekindling of a masterful precedent: bacteriophage

**DOI:** 10.3389/fcimb.2023.1202249

**Published:** 2023-05-01

**Authors:** Ghadir S. El-Housseiny, Mohamed H. Al-Agamy, Khaled M. Aboshanab

**Affiliations:** ^1^ Department of Microbiology and Immunology, Faculty of Pharmacy, Ain Shams University, Cairo, Egypt; ^2^ Department of Pharmaceutics, College of Pharmacy, King Saud University, Riyadh, Saudi Arabia; ^3^ Department of Microbiology and Immunology, Faculty of Pharmacy, Al-Azhar University, Cairo, Egypt

**Keywords:** bacteriophage, MRSA, antimicrobial resistance, phage therapy, optimization

## Introduction

Our world is facing a real menace because of antibiotic resistance, which, according to the World Health Organization, is one of the top 10 global threats to health. It is anticipated that approximately 10 million annual fatalities attributed to drug-resistant infections will befall by 2050, (Tangcharoensathien et al., 2017). To tackle such a risk, an innovative tactic is needed. The rekindling of a Masterful Precedent: Bacteriophage is a Research Topic in Frontiers in Cellular and Infection Microbiology aimed at spotlighting the potential of using the tiny but proficient creatures, bacteriophages, as therapeutic antibacterial agents. The four publications, three original research articles and one review, in this collection highlight the efficacy of these bacteriophages and emphasize their role in the everlasting war against bacterial resistance. We conclude by pinpointing unresolved queries with the aim of inspiring novel investigations in the future.

## Promising bacteriophages

Among the resistant bacteria, methicillin-resistant *Staphylococcus aureus* (MRSA) is a precarious multi-drug resistant (MDR) organism that causes infections as tough as pneumonia, skin, soft tissue, and diabetic foot infections (MRSA|CDC, 2019). *Staphylococcus aureus* has been affirmed by The Infectious Diseases Society of America (IDSA) as one of the lethal “ESKAPE” microorganisms, indicating its aptitude to escape even the last hope antibiotics, for instance, vancomycin (Santajit and Indrawattana, 2016). Hence, the work done by Abd-Allah et al. aimed at obtaining promising anti-MRSA phages. A total of five phages were separated from diverse sources like chicken egg rinses, raw milk, and, bizarrely, raw chicken and fish rinses. The phage obtained from raw fish rinse was lytic on all the 23 MRSA isolates collected from various clinical specimens and, hence, was selected for further studies. Based on electron microscopy, this phage was proposed to be of the Siphoviridae family, order Caudovirales. Upon testing its steadiness, it was found to display reasonable thermal stability and viability at different pH levels and good stability against all assessed viricidal organic solvents and UV light. This suggests its potential as an anti-MRSA tool in pharmaceutical formulations. Statistical optimization by response surface methodology (RSM) was designed for maximizing phage production. The ideal conditions advised by the created model were a pH of seven, sucrose of 0.5% w/v, peptone of 0.1% w/v, a temperature of 28°C, and a bacterial inoculum of 10^7^ CFU/ml, which enhanced the phage titer by a 2-log fold. Taken together, these results point out the phage’s lytic capability on MRSA.

The other study carried out by Abd-Allah et al. also targeted the optimization of anti-MRSA bacteriophage production but this time originating from the raw chicken rinse. This phage was believed to fit with Podoviridae, order Caudovirales, and survived a range of extreme states, and its production was optimized by the D-optimal design *via* RSM. The optimal conditions that yielded a 2-log fold rise in the titer were pH 8, glycerol 0.9% v/v, peptone 0.08% w/v, and 10^7^ CFU/ml as the host inoculum size. Once more, the findings proved the phage’s efficacy against the multidrug-resistant bacterium, MRSA.

Although both studies carried out by Abd-Allah et al. dealt with production optimization and stability tests on anti-MRSA phages, the source of the bacteriophages was different in each case, which led to phages belonging to different families as clarified. Moreover, the sources for phages are usually chosen based on the possibility of incorporating a certain host and, hence, bacteriophages specific against it. Known *S. aureus* bacteriophages are usually isolated from sewage; however, these two studies are some of the few that report *S. aureus* phage isolation from fish or chicken.

The adeptness of *S. aureus* to resist a broad array of antibiotics renders it a perfect model for phage therapy studies. Bacteriophage therapy tends to be more focused and overcomes resistance development. Nevertheless, numerous hindrances remain, chiefly concerning their action *in vivo*, demanding the use of animal models to evaluate phage efficacy. Therefore, Plumet et al. provided a review of the work already accomplished, stretching from case reports to clinical trials on diverse animal models. They found that both invertebrate and vertebrate animal models were established, which enhance our perception of the phage therapy processes on living organisms. The review deduced that there are only a few bacteriophage treatments for numerous infections in case studies, and a few clinical trials comprising different therapy approaches toward *S. aureus* infections have been reported. Remarkably, most studies reviewed concluded that staphylococcal phages treatment was most successful when applied in combination with antibiotics, approving phage therapy as potential unconventional management.

While the previous three studies focused on MDR *Staphylococcus aureus* phages, the former two discussed production optimization and the latter summarized clinical trials and established animal models; the last study in this collection was meant to fill the gap in *Klebsiella pneumoniae* phages and their characterization. *Klebsiella pneumoniae* is another prominent cause of death whose MDR strains are labeled as a universal human hazard, demanding the search for unconventional remedies. In their study, Zaki et al. targeted the discovery of specific anti-MDR *K. pneumoniae* phages. Phage vB_Kpn_ZCKp20p had the widest host range out of six phages obtained from urban and medical sewage and was consequently fully characterized. Transmission electron microscopy suggested the tailed phage to be of the Siphoviridae family. Evaluation *in vitro* showed great lytic activity of a 30 min latent period and burst size of ∼100 PFU/cell. Stability at temperatures reaching 70°C and pH ranges from 2 to 12 was revealed. Furthermore, it possessed antibiofilm activity and was safe on human skin fibroblasts. Whole genome sequencing and annotation revealed that out of 85 expected genes, 1 tRNA gene and 33 genes encoded proteins with allocated roles. Phage vB_Kpn_ZCKp20p was found to belong to the same genus as *Klebsiella* phages ZCKP8 and 6691 but most probably represents a new species as predicted by comparative genomics and phylogenetic analysis. Hence, Phage vB_Kpn_ZCKp20p is an original phage with prospects to be used against biofilm-forming *K. pneumoniae* and could be a potential source for antibacterial and antibiofilm products, which will be independently investigated in upcoming research.

## Future perspectives

Jointly, the articles collected on this Research Topic contribute significantly to our understanding of phage therapy. Nonetheless, essential questions linger around this dynamic topic.

Although different works of literature reported so far support the belief that phage therapy may be very useful to treat *S. aureus* infections when used in conjunction with antibiotics, only limited publications exist, and additional work is necessary to fully comprehend the dynamics in combination therapy to be excellently utilized in clinical practice. Moreover, a substantial gap in knowledge remains about the usage, practicality, and safety of the several human routes of administration. Recognizing phage pharmacology including pharmacokinetics and pharmacodynamics is also vital for its utility in healthcare situations; however, to date, no such research is reported in existing works of literature. Another inadequacy of the existing research is the absence of a proven and structured procedure for phage extraction and purification which results in variations in the results of the different studies described. In conclusion, promising phages must undergo several testing and well-planned clinical trials to achieve profits from bacteriophages as proficient antibacterial therapeutic tools.

## Author contributions

GE-H wrote the manuscript. All authors listed have made a substantial contribution to the work and approved it for publication.
